# Association of psoriasis with allergic multimorbidity of asthma, rhinitis, and eczema among adolescents: a cross-sectional study

**DOI:** 10.1186/s13223-024-00907-6

**Published:** 2024-07-24

**Authors:** Ali H. Ziyab, Yaser Ali, Dina Zein, Manal Al-Kandari, John W. Holloway, Wilfried Karmaus

**Affiliations:** 1 Department of Community Medicine and Behavioral Sciences, College of Medicine, Kuwait University, P. O. Box 24923, Safat, 13110 Kuwait; 2 Department of Internal Medicine, Mubarak Al-Kabeer Hospital, Ministry of Health, Jabriya, Kuwait; 3 Department of Public Health Policy and Management, School of Global Public Health, New York University, NY, USA; 4 Human Development and Health, Faculty of Medicine, University of Southampton, Southampton, UK; 5 Division of Epidemiology, Biostatistics and Environmental Health, School of Public Health, University of Memphis, Memphis, TN USA

**Keywords:** Psoriasis, Eczema, Asthma, Rhinitis, Multimorbidity, Adolescents

## Abstract

**Background:**

Associations between psoriasis and allergic diseases (asthma, rhinitis, and eczema) in children have been reported in a limited number of studies, and the association between psoriasis and multimorbidity (co-occurrence) of allergic diseases remains unclear. Hence, this study aimed to assess the association between psoriasis and the co-occurrence of asthma, rhinitis, and eczema in adolescents.

**Methods:**

This school-based cross-sectional study enrolled adolescents (*n* = 3,864) aged 11–14 years. Parents completed a questionnaire on doctor-diagnosed psoriasis as well as symptoms and clinical history of asthma, rhinitis, and eczema. Eight nonoverlapping groups comprising single and co-occurring current (past 12 months) asthma, rhinitis, and eczema were identified. A multinomial logistic regression model was used to estimate the adjusted odds ratios (aOR) and 95% confidence intervals (CI).

**Results:**

In the analytical sample (*n* = 3,710; 1,641 male and 2,069 female participants), 3.5% reported doctor-diagnosed psoriasis, and 15.7%, 15.0%, and 10.3% had current asthma, rhinitis, and eczema symptoms, respectively. Doctor-diagnosed psoriasis was associated with “asthma only” (aOR = 2.11, 95% CI: 1.15–3.89), “eczema only” (6.65, 4.11–10.74), “asthma + eczema” (5.25, 2.36–11.65), “rhinitis + eczema” (3.60, 1.07–12.15), and “asthma + rhinitis + eczema” (7.38, 2.93–18.58). Doctor-diagnosed psoriasis was not statistically significantly associated with “rhinitis only” (1.42, 0.71-–2.84) and “asthma + rhinitis” (1.78, 0.69–4.56).

**Conclusion:**

Our findings indicate that psoriasis is associated with the co-occurrence of allergic diseases among adolescents. However, further studies are required to investigate which biological mechanisms may be shared between psoriasis and allergic diseases.

## Introduction

Psoriasis is a chronic, immune-mediated inflammatory skin disease that affects over 60 million people worldwide, with varying prevalence estimates across regions [1]. The systemic impact of psoriasis-related inflammation causes long-term damage to multiple tissues and organs [2]. Patients with psoriasis may present different comorbidities, including psoriatic arthritis, metabolic syndrome (obesity, hypertension, type 2 diabetes, and dyslipidemia), cardiovascular disease (stroke and myocardial infarction), chronic obstructive pulmonary disease, chronic kidney disease, and inflammatory bowel disease [1, 2]. In addition, mental disorders, including depression and anxiety, have been reported as common comorbidities in patients with psoriasis [1, 2]. Hence, psoriasis impairs the physical and psychosocial well-being of affected individuals.

Although the pathophysiology of psoriasis remains unclear, cross-talk between innate and adaptive immune systems underlies the inflammatory infiltrate observed in psoriasis [3]. Activated myeloid dendritic cells release interleukin-12 (IL-12) and IL-23, which induce the proliferation of T-helper type 1 (Th1), Th17, and Th22 cells that subsequently produce pro-inflammatory cytokines (e.g., IL-17, IL-122, interferon gamma [IFN-γ], and tumor necrosis factor-alpha [TNF-α]) that characterize psoriasis, with the IL-23/Th17 pathway being the most predominant [2]. In contrast, allergic diseases, such as asthma, rhinitis, and eczema (atopic dermatitis), mainly involve skewed Th2-cells response to foreign bodies (allergens), which can lead to over-expression of IL-4 and IL-13, and subsequently increased production of immunoglobulin E, thereby causing an allergic reaction [4]. Although up-regulation of Th1 cells has been hypothesized to correlate with down-regulation of Th2 cells and vice versa, this paradigm has been challenged as the coexistence of autoimmune diseases caused by Th1 immune responses and allergic diseases caused by Th2 immune responses is not rare [5, 6]. Furthermore, genetic studies on allergic and autoimmune diseases have demonstrated considerable commonality in susceptibility loci [7], especially between eczema and psoriasis as inflammatory diseases of the skin [8], while others have reported opposing genetic susceptibility at the same loci [9].

The association between psoriasis and allergic diseases has been assessed in a limited number of studies. Majority of the studies that have assessed the association between psoriasis and allergic diseases have reported positive associations and suggested that psoriasis and allergic diseases have a common pathogenesis [10–15], while few reports found no or inverse association between psoriasis and allergic diseases [16, 17]. Given that the multimorbidity (co-occurrence) of allergic disease is not rare [18], the association between psoriasis and multimorbidity of allergic diseases requires further investigations. Hence, we aimed to evaluate the association of psoriasis with asthma, rhinitis, and eczema both as single and co-occurring conditions in adolescents.

## Methods

### Study design, setting, and population

This school-based cross-sectional study enrolled adolescents (*n* = 3,864) aged 11–14 years resident in Kuwait. As previously described [19], a representative sample of middle school students attending public schools was obtained using stratified two-stage cluster sampling. The study questionnaire was sent home with the adolescents for parental/guardian completion and return. Ethical approval for the current study was obtained from the Standing Committee for the Coordination of Health and Medical Research, Ministry of Health, Kuwait (No. 2016/451). Written informed consent was obtained from the parents or legal guardians of the adolescents participating in this study, which was conducted following the principles and guidelines of the Declaration of Helsinki for medical research involving human participants.

### Ascertainment of study variables

Ever doctor-diagnosed psoriasis was determined by an affirmative response by the parent/guardian to the following question: “Has this child ever been diagnosed with psoriasis by a doctor?” [20]. The International Study of Asthma and Allergies in Childhood (ISAAC) methodology was applied to ascertain allergic diseases [21]. Current (past 12 months) eczema was defined as “ever doctor-diagnosed eczema” and/or “having ever had a recurrent itchy rash for at least 6 months” plus “having an itchy rash at any time in the past 12 months that affected the folds of the elbows or the areas behind the knees, in front of the ankles, under the buttocks, or around the neck, ears, or eyes” [22, 23]. Current asthma was defined by an affirmative response to the items “history of physician-diagnosed asthma” and “wheezing in the past 12 months” and/or “asthma treatment in the past 12 months” [19, 24]. Current rhinitis was defined as “ever doctor-diagnosed rhinitis” and “having problems with a sneezing, runny, or blocked nose in the absence of a cold or flu in the past 12 months” [25]. Combinations of current asthma, rhinitis, and eczema resulted in eight nonoverlapping groups of single and coexisting allergic diseases: “no allergic disease,” “asthma only,” “rhinitis only,” “eczema only,” “asthma + rhinitis,” “asthma + eczema,” “rhinitis + eczema,” and “asthma + rhinitis + eczema” groups.

### Covariates

The parent/guardian reported the mode of participant birth (vaginal or cesarean section) and whether the participant was ever directly fed at the breast during infancy. Household exposure to secondhand smoke was assessed by asking whether any household member smoked cigarettes or tobacco-related products inside the home. To ascertain exposure to household cats and dogs during infancy, two separate questions were asked: “Did you have a cat/dog in your home during the first year of this child life?”

### Statistical analysis

Analyses were conducted using SAS 9.4 (SAS Institute, Cary, NC, USA). The statistical significance level was set at α = 0.05. Descriptive analyses were performed to calculate frequencies and proportions of categorical variables. Chi-square (*X*^2^) test was used to assess associations between categorical variables. The association between psoriasis status (exposure variable) and allergic diseases (outcome variable) was assessed using: (i) binary logistic regression when evaluating the association with each allergic disease (non-mutually exclusive), and (ii) multinomial logistic regression when evaluating the association with the single/co-occurring allergic disease(s) [mutually exclusive; nominal outcome variable, with the “no allergic disease” category set as the reference]. Adjusted odds ratios (aOR) and their 95% confidence intervals (CI) were estimated. In addition to age and sex, individual characteristics that demonstrated possible association (*p*-value < 0.2) with psoriasis were included in the multivariable models as potential confounders.

## Results

A total of 5,228 schoolchildren (2,483 male and 2,745 female adolescents) were invited to participate, of whom 3,864 (1,695 male and 2,169 female adolescents) agreed to participate (response proportion: 73.9%). The analytical sample (*n* = 3,710; restricted to participants with complete information on ever doctor-diagnosed psoriasis, current asthma, rhinitis, and eczema) and the total study sample (*n* = 3,864) were similar regarding the studied characteristics (Table 1). Of the total analytical sample, 3.5% of the adolescents reported having doctor-diagnosed psoriasis. The prevalence of current (past 12 months) asthma, rhinitis, and eczema were estimated to be 15.7%, 15.0, and 10.3%, respectively (Table 1).

**Table 1:** Characteristics of the total study sample and the analytical study sample

Variables	Total study sample (*n* = 3864)	Analytical study sample^*^ (*n* = 3710)
**Sex, ** *n* ** (%)**		
Male	1695 (43.9)	1641 (44.2)
Female	2169 (56.1)	2069 (55.8)
**Age (years)**,** n (%)**		
≤ 11	1065 (27.6)	1026 (27.6)
12	1170 (30.3)	1125 (30.3)
13	964 (24.9)	919 (24.8)
≥ 14	665 (17.2)	640 (17.3)
**BMI-for-age groups**,** n (%)**		
Thinness (< -2 SD)	219 (5.8)	209 (5.8)
Normal (-2 to 1 SD)	1517 (40.1)	1457 (40.1)
Overweight (> 1 to 2 SD)	961 (25.3)	921 (25.3)
Obese (> 2 SD)	1089 (28.8)	1048 (28.8)
Missing, n	78	75
**Mode of birth**,** n (%)**		
Vaginal	3106 (81.8)	2998 (81.7)
Cesarean section	692 (18.2)	673 (18.3)
Missing, n	66	39
**Breastfeeding ever**,** n (%)**		
Yes	2894 (76.3)	2796 (76.3)
Missing, n	72	45
**Secondhand smoke exposure**,** n (%)**		
Yes	1755 (45.8)	1694 (45.8)
Missing, n	28	8
**Cat exposure in infancy**,** n (%)**		
Yes	232 (6.1)	224 (6.1)
Missing, n	35	15
**Dog exposure in infancy**,** n (%)**		
Yes	85 (2.2)	84 (2.3)
Missing, n	32	10
**Ever doctor-diagnosed psoriasis**		
Yes	136 (3.6)	131 (3.5)
Missing, n	58	0
**Current eczema**,** n (%)**		
Yes	388 (10.2)	381 (10.3)
Missing, n	73	0
**Current asthma**,** n (%)**		
Yes	600 (15.7)	581 (15.7)
Missing, n	35	0
**Current rhinitis**,** n (%)**		
Yes	566 (15.1)	558 (15.0)
Missing, n	105	0

BMI: body mass index; SD: standard deviation

^*^ Refers to the sample of participants with complete information on psoriasis status, current eczema status, current, asthma status, and current rhinitis status

Table 2 shows the prevalence of ever doctor-diagnosed psoriasis according to individual characteristics. The prevalence of psoriasis was similar in male and female participants (3.4% vs. 3.7%, *p* = 0.598). The prevalence of ever doctor-diagnosed psoriasis was higher among adolescents who were exposed to secondhand smoke in their households than among those who were not exposed (4.3% vs. 2.9%, *p* = 0.019). Similarly, having cats during infancy was associated with an increased prevalence of ever doctor-diagnosed psoriasis (7.6% vs. 3.3%, *p* < 0.001). Breastfed during infancy was associated with a lower prevalence of ever doctor-diagnosed psoriasis compared with those who were never breastfed (3.1% vs. 4.8%, *p* = 0.014).

**Table 2:** Prevalence of ever-doctor diagnosed psoriasis according to individual characteristics

Variables	*n*	Ever doctor-diagnosed psoriasis, % (*n*)	*P*-value^*^
**Sex**			
Male	1641	3.4 (55)	0.598
Female	2069	3.7 (76)	
**Age (years)**			
≤ 11	1026	3.7 (38)	0.245
12	1125	2.7 (30)	
13	919	4.2 (39)	
≥ 14	640	3.8 (24)	
**BMI-for-age groups**			
Thinness (< -2 SD)	209	6.2 (13)	0.145
Normal (-2 to 1 SD)	1457	3.4 (50)	
Overweight (> 1 to 2 SD)	921	3.9 (36)	
Obese (> 2 SD)	1048	3.1 (32)	
**Mode of birth**			
Vaginal	2998	3.6 (107)	0.848
Cesarean section	673	3.4 (23)	
**Breastfeeding ever**			
Yes	2796	3.1 (86)	0.014
No	869	4.8 (42)	
**Secondhand smoke exposure**			
Yes	1694	4.3 (73)	0.019
No	2008	2.9 (58)	
**Cat exposure in infancy**			
Yes	224	7.6 (17)	< 0.001
No	3471	3.3 (114)	
**Dog exposure in infancy**			
Yes	84	6.0 (5)	0.226
No	3616	3.5 (126)	

BMI: body mass index; SD: standard deviation

^*^ Calculated using chi-square (*X*^2^) test

Associations between ever doctor-diagnosed psoriasis and non-mutually exclusive occurrence of current asthma, rhinitis, and eczema are shown in Table 3. Ever doctor-diagnosed psoriasis was associated with increased prevalence of current asthma (aOR = 1.93, 95% CI: 1.28–2.91) and current eczema (aOR = 5.36, 95% CI: 3.68–7.82), but not current rhinitis (aOR = 1.31, 95% CI: 0.83–2.07; Table 3).

**Table 3:** Adjusted associations between ever doctor-diagnosed psoriasis and allergic diseases

	Ever doctor-diagnosed psoriasis			
	Yes (*n* = 131)	No (*n* = 3579)		
Allergic disease	% (*n*)	% (*n*)	aOR^‡^ (95% CI)	*P*-value
Asthma	26.0 (34)	15.3 (547)	1.93 (1.28–2.91)	0.002
Rhinitis	18.3 (24)	14.9 (534)	1.31 (0.83–2.07)	0.253
Eczema	36.6 (48)	9.3 (333)	5.36 (3.68–7.82)	< 0.001

aOR: Adjusted odds ratio; CI: Confidence interval

^‡^ Adjusted for sex, age, BMI-for-age groups, breastfeeding, secondhand smoke exposure, and cat exposure in infancy

Associations between ever doctor-diagnosed psoriasis and single and co-occurring allergic diseases are shown in Table 4. The prevalence of “asthma only” (aOR = 2.11, 95% CI: 1.15–3.89) and “eczema only” (aOR = 6.65, 95% CI: 4.11–10.74), but not the prevalence of “rhinitis only” (aOR = 1.42, 95% CI: 0.71–2.84), was increased in children with ever doctor-diagnosed psoriasis compared to those without prior diagnosis of psoriasis. Moreover, ever doctor-diagnosed psoriasis was associated with the co-occurrence of “asthma + eczema” (aOR = 5.25, 95% CI: 2.36–11.65), “rhinitis + eczema” (aOR = 3.60, 95% CI: 1.07–12.15), and “asthma + rhinitis + eczema” (aOR = 7.38, 95% CI: 2.93–18.58; Table 4).

**Table 4:** Adjusted associations between ever doctor-diagnosed psoriasis and single and co-occurring allergic diseases

		Ever doctor-diagnosed psoriasis			
	Total (*n* = 3710)	Yes (*n* = 131)	No (*n* = 3579)		
Allergic disease	% (*n*)	% (*n*)	% (*n*)	aOR^‡^ (95% CI)	*P*-value
None	68.1 (2526)	40.5 (53)	69.1 (2473)	1.00 (Reference)	–
Asthma only	8.9 (328)	11.5 (15)	8.8 (313)	2.11 (1.15–3.89)	0.016
Rhinitis only	9.0 (334)	7.6 (10)	9.1 (324)	1.42 (0.71–2.84)	0.316
Eczema only	6.1 (227)	23.7 (31)	5.5 (196)	6.65 (4.11–10.74)	< 0.001
Asthma + Rhinitis	3.8 (141)	3.8 (5)	3.8 (136)	1.78 (0.69–4.56)	0.232
Asthma + Eczema	1.9 (71)	6.1 (8)	1.8 (63)	5.25 (2.36–11.65)	< 0.001
Rhinitis + Eczema	1.1 (42)	2.3 (3)	1.1 (39)	3.60 (1.07–12.15)	0.039
Asthma + Rhinitis + Eczema	1.1 (41)	4.6 (6)	1.0 (35)	7.38 (2.93–18.58)	< 0.001

aOR: Adjusted odds ratio; CI: Confidence interval

^‡^ Adjusted for sex, age, BMI-for-age groups, breastfeeding, secondhand smoke exposure, and cat exposure in infancy

## Discussion

In this study, we demonstrated that doctor-diagnosed psoriasis was associated with an increased prevalence of current asthma and eczema, but not with rhinitis. Moreover, when assessing the mutually exclusive occurrence (nonoverlapping groups) of allergic diseases, psoriasis was associated with “asthma only” and “eczema only,” and the co-occurrence of “asthma + eczema,” “rhinitis + eczema,” and “asthma + rhinitis + eczema”. These findings suggest that the association between psoriasis and asthma is not dependent on the coexistence of other allergic diseases (i.e., eczema and rhinitis). Moreover, we demonstrated that psoriasis increased the odds of co-occurring allergic diseases.

In agreement with our findings, previous studies have found associations between psoriasis and asthma [10–14, 26]. Moreover, the association observed between psoriasis and eczema is supported by previous reports, as summarized in a meta-analysis [27]. To the best of our knowledge, only two prior studies have investigated such associations among children and reported increased odds of asthma [13, 15] and eczema [15] in relation to psoriasis. Although we observed an increased odds of rhinitis in relation to psoriasis (aOR = 1.31, 95% CI: 0.83–2.07), this association was not statistically significant, contradicting previous findings that observed increased risk of rhinitis related to psoriasis in children [13, 15]. Our analysis adds to the literature by investigating associations between psoriasis and the coexistence of asthma, rhinitis, and eczema. This investigation showed that psoriasis was associated with increased odds of single and co-occurrence of allergic diseases, with the odds of the co-occurrence of “asthma, rhinitis, and eczema” being noticeably increased. Hence, our findings suggest that psoriasis and allergic diseases are not mutually exclusive and may share certain biological mechanisms.

Classically, psoriasis and allergic diseases are considered Th1- and Th2-driven diseases, respectively. Nonetheless, the role of the IL-23/Th17 pathway leading to IL-17 secretion has emerged as dominant in the pathophysiology of psoriasis [1, 2]. Moreover, a recent study has shown that a group of patients with asthma had high IL-17 levels and demonstrated psoriasis-like immunophenotypic features [28]. A study using murine models has shown that psoriatic inflammation enhanced airway inflammation through the IL-23/Th17 axis [29]. Similarly, IL-17 reportedly contributes to the immune dysregulation observed in patients with eczema [30]. Therefore, Th17-cell activation leading to IL-17 production could be a common link between psoriasis and allergic diseases. In addition, a genome-wide association study demonstrated overlapping loci between eczema, asthma, and psoriasis, further indicating a shared genetic background [8].

Moreover, in terms of risk factors, genetic predisposition is the major contributor to the development of psoriasis, with few environmental and behavioral/lifestyle factors being implicated in the etiology of psoriasis [31, 32]. Alcohol use, smoking, and obesity have been shown to be risk factors for psoriasis [33–35]. To investigate possible causal effects of the identified risk factors, Mendelian Randomization studies have reported potential causal effects of smoking and obesity on psoriasis, but not alcohol consumption [36–38]. Similarly, smoking and obesity have been shown to be risk factors for the development of allergic diseases [39–41]. Moreover, breastfeeding has been shown to be associated with allergic diseases [42, 43], and more recently few studies have reported the association between breastfeeding and psoriasis [20, 44]. Collectively, the aforementioned factors have been speculated to alter immune development and responses, hence altering the risk of immune mediated diseases such as psoriasis and allergic diseases.

The population-based design (capturing the spectrum of diseases) and large sample size are the strengths of our study. Although the reliability (Cohen’s kappa: 0.7558) [45] and validity (sensitivity: 56%; specificity: 99%; positive predictive value: 78%; negative predictive value: 96%) [46] of self-reported psoriasis diagnoses are reportedly high, misclassification of the disease status cannot be eliminated, which can bias the estimated measures of association. Our assessment focused on doctor-diagnosed psoriasis reported by parent/guardian. A prior study showed that the inclusion of “self-reported doctor-diagnosed psoriasis” compared to only “self-reported psoriasis” yielded increased validity [46]. Moreover, we have previously shown that the majority of individuals who reported a doctor-diagnosed psoriasis in our study sample have reported the involvement of typical psoriasis anatomical sites (scalp [47.6%], and extensor surfaces of the knees [50%] and elbows [38.1%]) [20], which further supports the validity of our definition of psoriasis. Similarly, the misclassification of asthma, rhinitis, and eczema symptoms cannot be excluded. Nonetheless, we have used the standardized ISAAC questionnaire to ascertain allergic diseases, which has been shown to have good validity [21]. For instance, a prior study showed that the ISAAC criteria for ascertaining asthma symptoms as compared to physician diagnosis of asthma had a sensitivity of 85% and specificity of 81% [47]. Similarly, among children in the UK, using GP-recorded asthma as the gold standard, parental-reported ever doctor-diagnosed asthma had a sensitivity of 88.5% and a specificity of 95.7% [48]. Moreover, prior studies that defined asthma in a similar manner to our “current asthma” variable showed that their defined asthma to be associated with reduced lung function parameters [24, 49]. Compared to prior studies that defined asthma in a similar manner to our study, the estimated prevalence of current asthma (15.7%) in this report is similar to the prevalence estimate of current asthma (16.1%) in a preceding study conducted among adolescents aged in 16–19 years in Kuwait [50], and comparable to current asthma prevalence estimate among 18-year old Isle of Wight birth cohort participants (17.7%) in the UK [51]. Such observations further support the used asthma definition. Furthermore, given that the magnitude and direction of the estimated measures of association (ORs) in our study are similar to that in previous studies [10, 13–15, 26], we speculate that the effect of misclassification, if any, should be minimal on our results. It is essential to also indicate that our reported cross-sectional (concurrent) associations do not implicate any causal associations.

## Conclusions

This study demonstrated associations between psoriasis and the single- and co-occurrence of allergic diseases in a sample of adolescents, and these findings add new knowledge into the limited literature in this area. Such associations further suggest that the coexistence of psoriasis and allergic diseases is not rare, and the paradigm of their nonoverlapping existence requires reexamination. Further studies are needed to investigate the biological mechanisms and genetic polymorphisms shared by psoriasis and allergic diseases that may result in their coexistence. The elucidation of these mechanisms may improve the clinical management of patients with coexisting psoriasis and allergic diseases.

## Data Availability

The datasets used and analyzed during the current study are available from the corresponding author on reasonable request.

## Abbreviations

IL: interleukin
Th: T-helper type cells
IFN-γ: interferon gamma
TNF-α: tumor necrosis factor-alpha
aOR: adjusted odds ratio
CI: confidence interval

## References

[1] Griffiths CEM, Armstrong AW, Gudjonsson JE, Barker J, Psoriasis. Lancet. 2021;397:1301–15. 33812489 10.1016/S0140-6736(20)32549-6

[2] Armstrong AW, Read C, Pathophysiology. Clinical presentation, and treatment of psoriasis: a review. JAMA. 2020;323:1945–60. 32427307 10.1001/jama.2020.4006

[3] Gaspari AA. Innate and adaptive immunity and the pathophysiology of psoriasis. J Am Acad Dermatol. 2006;54:S67–80. 16488332 10.1016/j.jaad.2005.10.057

[4] Averbeck M, Gebhardt C, Emmrich F, Treudler R, Simon JC. Immunologic principles of allergic disease. J Dtsch Dermatol Ges. 2007;5:1015–28. 17976144 10.1111/j.1610-0387.2007.06538.x

[5] Simpson CR, Anderson WJ, Helms PJ, Taylor MW, Watson L, Prescott GJ, et al. Coincidence of immune-mediated diseases driven by Th1 and Th2 subsets suggests a common aetiology. A population-based study using computerized general practice data. Clin Exp Allergy. 2002;32:37–42. 12002734 10.1046/j.0022-0477.2001.01250.x

[6] Essl A, Loader D, Feldmann R, Steiner A, Sator P. Psoriasis and IgE-mediated allergy: correlation or mutual inhibition? A prospective cohort study in patients with mild or moderate to severe psoriasis. Wien Klin Wochenschr. 2021;133:997–1003. 32700084 10.1007/s00508-020-01683-0

[7] Shirai Y, Nakanishi Y, Suzuki A, Konaka H, Nishikawa R, Sonehara K, et al. Multi-trait and cross-population genome-wide association studies across autoimmune and allergic diseases identify shared and distinct genetic component. Ann Rheum Dis. 2022;81:1301–12. 35753705 10.1136/annrheumdis-2022-222460 PMC9380494

[8] Weidinger S, Willis-Owen SA, Kamatani Y, Baurecht H, Morar N, Liang L, et al. A genome-wide association study of atopic dermatitis identifies loci with overlapping effects on asthma and psoriasis. Hum Mol Genet. 2013;22:4841–56. 23886662 10.1093/hmg/ddt317 PMC3820131

[9] Baurecht H, Hotze M, Brand S, Buning C, Cormican P, Corvin A, et al. Genome-wide comparative analysis of atopic dermatitis and psoriasis gives insight into opposing genetic mechanisms. Am J Hum Genet. 2015;96:104–20. 25574825 10.1016/j.ajhg.2014.12.004 PMC4289690

[10] Fang HY, Liao WC, Lin CL, Chen CH, Kao CH. Association between psoriasis and asthma: a population-based retrospective cohort analysis. Br J Dermatol. 2015;172:1066–71. 25385450 10.1111/bjd.13518

[11] Egeberg A, Khalid U, Gislason GH, Mallbris L, Skov L, Hansen PR. Risk of psoriasis in patients with childhood asthma: a Danish nationwide cohort study. Br J Dermatol. 2015;173:159–64. 25801416 10.1111/bjd.13781

[12] Lonnberg AS, Skov L, Skytthe A, Kyvik KO, Pedersen OB, Meteran H, et al. Asthma in patients with psoriasis. Br J Dermatol. 2015;172:1660–1. 25533772 10.1111/bjd.13637

[13] Galili E, Barzilai A, Twig G, Caspi T, Daniely D, Shreberk-Hassidim R, et al. Allergic rhinitis and asthma among adolescents with psoriasis: a Population-based cross-sectional study. Acta Derm Venereol. 2020;100:adv00133. 32314795 10.2340/00015555-3485 PMC9137373

[14] Martin A, Thatiparthi A, Liu J, Ge S, Egeberg A, Wu JJ. Association between psoriasis and asthma among United States adults in the 2009–2014 National Health and Nutrition Examination Survey. J Am Acad Dermatol. 2022;86:709–12. 33882277 10.1016/j.jaad.2021.04.027

[15] Augustin M, Radtke MA, Glaeske G, Reich K, Christophers E, Schaefer I, et al. Epidemiology and comorbidity in children with psoriasis and atopic Eczema. Dermatology. 2015;231:35–40. 25966818 10.1159/000381913

[16] Kirsten N, Mohr N, Maul JT, Augustin M. Incidence of atopic conditions in people with psoriasis: a population-based analysis. Eur J Dermatol. 2021;31:60–4. 33648927 10.1684/ejd.2021.3963

[17] Landgren E, Braback L, Hedlin G, Hjern A, Rasmussen F. Psoriasis in Swedish conscripts: time trend and association with T-helper 2-mediated disorders. Br J Dermatol. 2006;154:332–6. 16433805 10.1111/j.1365-2133.2005.07004.x

[18] Haider S, Fontanella S, Ullah A, Turner S, Simpson A, Roberts G, et al. Evolution of Eczema, Wheeze, and Rhinitis from Infancy to Early Adulthood: four birth cohort studies. Am J Respir Crit Care Med. 2022;206:950–60. 35679320 10.1164/rccm.202110-2418OC PMC9802000

[19] Ziyab AH. Prevalence of food allergy among schoolchildren in Kuwait and its association with the coexistence and severity of asthma, rhinitis, and eczema: a cross-sectional study. World Allergy Organ J. 2019;12:100024. 30976380 10.1016/j.waojou.2019.100024 PMC6441753

[20] Ziyab AH, Karmaus W, AlShatti KA, Al-Kandari M, Hussein SH, Ali YM. Psoriasis among adolescents in Kuwait and the role of siblings, breastfeeding, and Household cat and secondhand smoke exposure: a cross-sectional study. Dermatol Ther (Heidelb). 2020;10:1137–53. 32844373 10.1007/s13555-020-00437-0 PMC7477028

[21] Asher MI, Keil U, Anderson HR, Beasley R, Crane J, Martinez F, et al. International Study of Asthma and allergies in Childhood (ISAAC): rationale and methods. Eur Respir J. 1995;8:483–91. 7789502 10.1183/09031936.95.08030483

[22] Odhiambo JA, Williams HC, Clayton TO, Robertson CF, Asher MI, Group IPTS. Global variations in prevalence of eczema symptoms in children from ISAAC Phase Three. J Allergy Clin Immunol. 2009;124:1251–e823. 20004783 10.1016/j.jaci.2009.10.009

[23] Hanifin JM, Rajka G. Diagnostic features of atopic dermatitis. Acta Derm Venereol Suppl (Stockh). 1980;92:44–7. 10.2340/00015555924447

[24] Arshad SH, Hodgekiss C, Holloway JW, Kurukulaaratchy R, Karmaus W, Zhang H et al. Association of Asthma and smoking with lung function impairment in adolescence and early adulthood: the Isle of Wight Birth Cohort Study. Eur Respir J. 2020;55. 10.1183/13993003.00477-2019 31831580

[25] Ziyab AH, Ali YM. Rhinoconjunctivitis among adolescents in Kuwait and Associated Risk factors: a cross-sectional study. Biomed Res Int. 2019;2019:3981064. 31815136 10.1155/2019/3981064 PMC6878814

[26] Joel MZ, Fan R, Damsky W, Cohen JM. Psoriasis associated with asthma and allergic rhinitis: a US-based cross-sectional study using the all of US Research Program. Arch Dermatol Res. 2023;315:1823–6. 36707438 10.1007/s00403-023-02539-z

[27] Cunliffe A, Gran S, Ali U, Grindlay D, Lax SJ, Williams HC, et al. Can atopic eczema and psoriasis coexist? A systematic review and meta-analysis. Skin Health Dis. 2021;1:e29. 35664974 10.1002/ski2.29 PMC9060081

[28] Ostling J, van Geest M, Schofield JPR, Jevnikar Z, Wilson S, Ward J, et al. IL-17-high asthma with features of a psoriasis immunophenotype. J Allergy Clin Immunol. 2019;144:1198–213. 30998987 10.1016/j.jaci.2019.03.027

[29] Nadeem A, Al-Harbi NO, Ansari MA, Al-Harbi MM, El-Sherbeeny AM, Zoheir KMA, et al. Psoriatic inflammation enhances allergic airway inflammation through IL-23/STAT3 signaling in a murine model. Biochem Pharmacol. 2017;124:69–82. 27984001 10.1016/j.bcp.2016.10.012

[30] Brunner PM, Guttman-Yassky E, Leung DY. The immunology of atopic dermatitis and its reversibility with broad-spectrum and targeted therapies. J Allergy Clin Immunol. 2017;139:S65–76. 28390479 10.1016/j.jaci.2017.01.011 PMC5405702

[31] Dand N, Mahil SK, Capon F, Smith CH, Simpson MA, Barker JN. Psoriasis and Genetics. Acta Derm Venereol. 2020;100:adv00030. 31971603 10.2340/00015555-3384 PMC9128944

[32] Di Meglio P, Villanova F, Nestle FO, Psoriasis. Cold Spring Harb Perspect Med. 2014;4. 10.1101/cshperspect.a015354 PMC4109580 25085957

[33] Brenaut E, Horreau C, Pouplard C, Barnetche T, Paul C, Richard MA, et al. Alcohol consumption and psoriasis: a systematic literature review. J Eur Acad Dermatol Venereol. 2013;27(Suppl 3):30–5. 23845150 10.1111/jdv.12164

[34] Armstrong AW, Harskamp CT, Dhillon JS, Armstrong EJ. Psoriasis and smoking: a systematic review and meta-analysis. Br J Dermatol. 2014;170:304–14. 24117435 10.1111/bjd.12670

[35] Aune D, Snekvik I, Schlesinger S, Norat T, Riboli E, Vatten LJ. Body mass index, abdominal fatness, weight gain and the risk of psoriasis: a systematic review and dose-response meta-analysis of prospective studies. Eur J Epidemiol. 2018;33:1163–78. 29680995 10.1007/s10654-018-0366-z PMC6290660

[36] Wei J, Zhu J, Xu H, Zhou D, Elder JT, Tsoi LC, et al. Alcohol consumption and smoking in relation to psoriasis: a mendelian randomization study. Br J Dermatol. 2022;187:684–91. 35764530 10.1111/bjd.21718

[37] Ogawa K, Stuart PE, Tsoi LC, Suzuki K, Nair RP, Mochizuki H, et al. A transethnic mendelian randomization study identifies causality of obesity on risk of Psoriasis. J Invest Dermatol. 2019;139:1397–400. 30528826 10.1016/j.jid.2018.11.023 PMC7028352

[38] Jin JQ, Elhage KG, Spencer RK, Davis MS, Hakimi M, Bhutani T, et al. Mendelian randomization studies in psoriasis and psoriatic arthritis: a systematic review. J Invest Dermatol. 2023;143:762–76. e3. 36822971 10.1016/j.jid.2022.11.014

[39] Xu S, Gilliland FD, Conti DV. Elucidation of causal direction between asthma and obesity: a bi-directional mendelian randomization study. Int J Epidemiol. 2019;48:899–907. 31005996 10.1093/ije/dyz070 PMC6659368

[40] Kantor R, Kim A, Thyssen JP, Silverberg JI. Association of atopic dermatitis with smoking: a systematic review and meta-analysis. J Am Acad Dermatol. 2016;75:1119–e251. 27542586 10.1016/j.jaad.2016.07.017 PMC5216172

[41] Saulyte J, Regueira C, Montes-Martinez A, Khudyakov P, Takkouche B. Active or passive exposure to tobacco smoking and allergic rhinitis, allergic dermatitis, and food allergy in adults and children: a systematic review and meta-analysis. PLoS Med. 2014;11:e1001611. 24618794 10.1371/journal.pmed.1001611 PMC3949681

[42] Lodge CJ, Tan DJ, Lau MX, Dai X, Tham R, Lowe AJ, et al. Breastfeeding and asthma and allergies: a systematic review and meta-analysis. Acta Paediatr. 2015;104:38–53. 26192405 10.1111/apa.13132

[43] Xue M, Dehaas E, Chaudhary N, O’Byrne P, Satia I, Kurmi OP. Breastfeeding and risk of childhood asthma: a systematic review and meta-analysis. ERJ Open Res. 2021;7. 10.1183/23120541.00504-2021 PMC8666625 34912884

[44] Das D, Thimjo J, Lebena A, Guo A, Enerback C, Ludvigsson J. Breast-feeding decreases the risk of developing psoriasis through early adulthood. Br J Dermatol. 2024. 10.1093/bjd/ljae043 PMC13077223 38305572

[45] Nymand LK, Andersen YMF, Thyssen JP, Egeberg A. Limitations of using questionnaires for assessing the prevalence of Psoriasis and atopic dermatitis among adults. JAMA Dermatol. 2021;157:971–7. 34232252 10.1001/jamadermatol.2021.2307 PMC8264750

[46] Modalsli EH, Snekvik I, Asvold BO, Romundstad PR, Naldi L, Saunes M. Validity of self-reported psoriasis in a General Population: the HUNT study, Norway. J Invest Dermatol. 2016;136:323–5. 26763455 10.1038/JID.2015.386

[47] Jenkins MA, Clarke JR, Carlin JB, Robertson CF, Hopper JL, Dalton MF, et al. Validation of questionnaire and bronchial hyperresponsiveness against respiratory physician assessment in the diagnosis of asthma. Int J Epidemiol. 1996;25:609–16. 8671563 10.1093/ije/25.3.609

[48] Cornish RP, Henderson J, Boyd AW, Granell R, Van Staa T, Macleod J. Validating childhood asthma in an epidemiological study using linked electronic patient records. BMJ Open. 2014;4:e005345. 24760357 10.1136/bmjopen-2014-005345 PMC4010849

[49] Karmaus W, Mukherjee N, Janjanam VD, Chen S, Zhang H, Roberts G, et al. Distinctive lung function trajectories from age 10 to 26 years in men and women and associated early life risk factors - a birth cohort study. Respir Res. 2019;20:98. 31118050 10.1186/s12931-019-1068-0 PMC6532227

[50] Alnajem A, Redha A, Alroumi D, Alshammasi A, Ali M, Alhussaini M, et al. Use of electronic cigarettes and secondhand exposure to their aerosols are associated with asthma symptoms among adolescents: a cross-sectional study. Respir Res. 2020;21:300. 33198741 10.1186/s12931-020-01569-9 PMC7670675

[51] Soto-Ramirez N, Ziyab AH, Karmaus W, Zhang H, Kurukulaaratchy RJ, Ewart S, et al. Epidemiologic methods of assessing asthma and wheezing episodes in longitudinal studies: measures of change and stability. J Epidemiol. 2013;23:399–410. 23994864 10.2188/jea.JE20120201 PMC3834276

